# Electrically Tunable Ultraflat Bands and π-Electron Magnetism in Graphene Nanoribbons

**DOI:** 10.1021/acs.jpclett.5c00121

**Published:** 2025-02-07

**Authors:** Ruize Ma, Nikita V. Tepliakov, Arash A. Mostofi, Michele Pizzochero

**Affiliations:** † Department of Physics, ETH Zürich, Zurich 8093, Switzerland; ‡ Departments of Materials and Physics, Imperial College London, London SW7 2AZ, United Kingdom; ¶ The Thomas Young Centre for Theory and Simulation of Materials, Imperial College London, London SW7 2AZ, United Kingdom; § Department of Physics, University of Bath, Bath BA2 7AY, United Kingdom; ∥ School of Engineering and Applied Sciences, Harvard University, Cambridge, Massachusetts 02138, United States

## Abstract

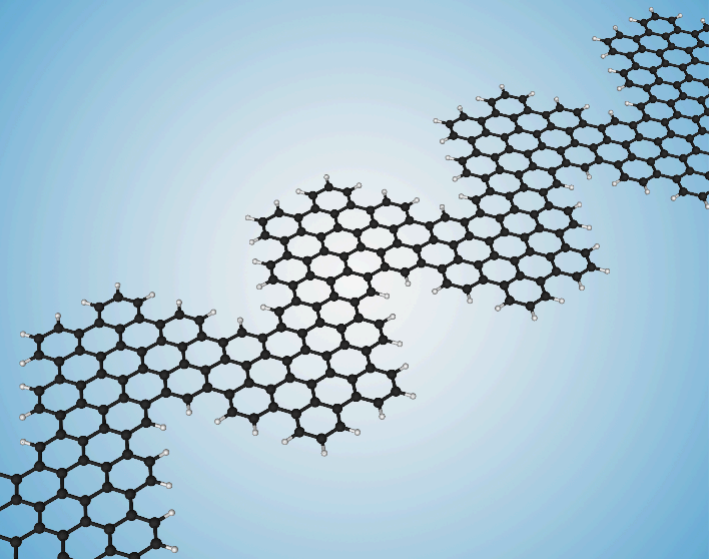

Atomically thin crystals hosting flat electronic bands have recently been identified as a rich playground for exploring and engineering strongly correlated phases. Yet, their variety remains limited, primarily to two-dimensional moiré superlattices. Here, we predict the formation of reversible, electrically induced ultraflat bands and π-electron magnetism in one-dimensional chevron graphene nanoribbons. Our *ab initio* calculations show that the application of a transverse electric field to these nanoribbons generates a pair of isolated, nearly perfectly flat bands with widths of approximately 1 meV around the Fermi level. Upon charge doping, these flat bands undergo a Stoner-like electronic instability, resulting in the spontaneous emergence of local magnetic moments at the edges of the otherwise nonmagnetic nanoribbon, akin to a one-dimensional spin-1/2 chain. Our findings expand the class of carbon-based nanostructures exhibiting flat bands and establish a novel route for inducing correlated electronic phases in chevron graphene nanoribbons.

*Introduction*. Two-dimensional nanostructures of graphene hosting flat electronic bands have emerged in the spotlight of condensed matter physics owing to their capacity to serve as platforms for accessing and manipulating a variety of quantum phases, with twisted bilayer graphene being the prime example.^1^ In twisted bilayer graphene, the introduction of a moiré interference pattern through a relative twist angle between a pair of stacked layers leads to a periodic modulation of the lattice potential. In the vicinity of the “magic” twist angle of 1.1°, this results in the formation of flat bands at the Fermi level,^2−4^ the hallmark of strong electron–electron interactions. These flat bands support a wide spectrum of unconventional electronic phases, most notably superconductivity,^5^ correlated insulator behavior,^6^ and magnetism,^7^ that are more often observed in bulk transition-metal oxides. Motivated by these findings, efforts to expand the family of graphene-based materials possessing flat bands have prompted the exploration of various strategies to impose spatially periodic moiré potentials beyond twisting, e.g., through the application of lattice heterostrain^8^ or superlattice potential.^9^

In this Letter, we propose an alternative approach to achieve flat bands in graphene nanostructures *without* the introduction of a moiré pattern. To that end, we consider chevron graphene nanoribbons (CGNRs),^10,11^ a class of one-dimensional semiconductors composed of few-atom-wide strips of *sp*^2^-hybridized carbon atoms. Through *ab initio* calculations, we show that a transverse electric field induces a pair of isolated, ultraflat bands near the Fermi level, with bandwidths as narrow as 1 meV. Upon charge doping, these bands drive a magnetic phase transition in otherwise nonmagnetic chevron graphene nanoribbons, resulting in an array of localized magnetic moments analogous to a quantum spin chain. Our findings establish a novel mechanism for the realization of tunable flat bands and correlated phases beyond moiré materials, positioning these recently fabricated graphene nanoribbons as viable candidates to probe these intriguing phenomena.

*Electrically Induced Flat Bands in CGNR*. Our *ab initio* calculations are performed using spin-polarized density-functional theory (DFT). We apply the generalized gradient approximation to the exchange-correlation functional,^12^ as implemented in the SIESTA package.^13^ The Kohn–Sham wave functions of the valence electrons are represented as a linear combination of local basis functions of double-ζ plus polarization (DZP) quality in combination with a mesh cutoff of 400 Ry. Core electrons are replaced by norm-conserving pseudopotentials generated following the Troullier-Martins scheme.^14^ The integration over the one-dimensional Brillouin zone is carried out using a grid of 12 *k*-points. The atomic coordinates are optimized at zero electric field and charge neutrality, with a tolerance for atomic forces of 0.01 eV/Å. Vacuum regions of 20 Å are introduced in the two nonperiodic directions in order to avoid spurious interactions between periodic replicas.

We begin by examining the electronic structure of the CGNR depicted in Figure 1a, a representative member of this class of nanoribbons that has been recently synthesized in atomically precise fashion via solution^15^ and on-surface techniques.^10,16^ The CGNR exhibits a nonmagnetic ground state. The band structure, given in Figure 1b, indicates a sizable, direct band gap located at the center of the Brillouin zone, with the valence and conduction bands possessing bandwidths of 186 and 144 meV, respectively. In Figure 1c,d, we show the local density of states pertaining to the band edges, which are found to be delocalized and evenly distributed across the nanoribbon.

**Figure 1:**
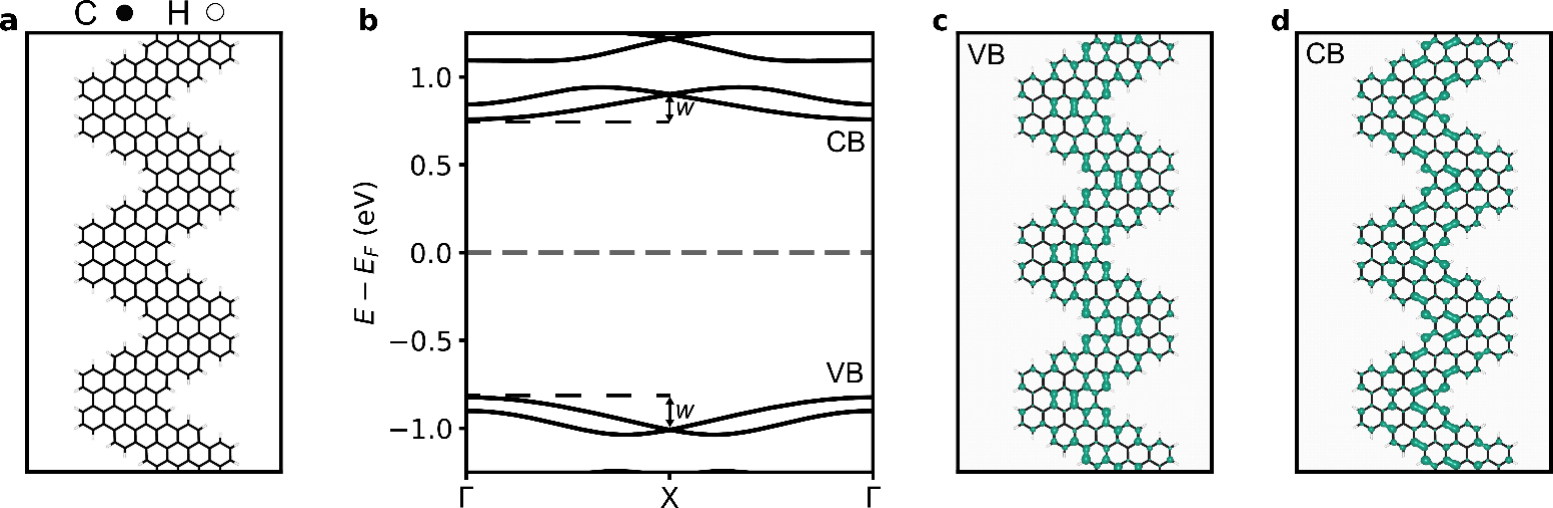
**Electronic structure of CGNR. a**, Atomic structure of a chevron graphene nanoribbon (CGNR). Carbon and hydrogen atoms are colored black and white, respectively. **b**, Electronic band structure of the CGNR. Also indicated are the valence band (VB) and conduction band (CB), along with the definition of bandwidth (*w*). The energy is referenced to the Fermi level (*E*_*F*_), marked by a horizontal dashed line. Local density of states (LDOS), i.e., the density of states weighted by the amplitude of the corresponding wave functions integrated between the energy interval encompassing the **c**, VB and **d**, CB.

Next, we explore the effect of an external electric field on the CGNR. We impose the field in the in-plane direction orthogonal to the periodicity of the nanoribbon, as schematically illustrated in Figure 2a. This transverse electric field has a significant effect on the electronic structure of the CGNR. Similar to the behavior predicted in armchair-^17^ and zigzag-edged graphene nanoribbons,^18^ increasing the strength of the field narrows the band gaps by more than 85%, as seen in Figure 2b. Interestingly, Figure 2c shows that the field reduces both the valence and conduction bandwidths, albeit in a nonmonotonic fashion. Ultimately, the band structure transitions into that given in Figure 2d, where a pair of completely isolated, ultraflat bands symmetrically centered at the Fermi level arises. The evolution of the band structure with the strength of the field is provided in Supporting Information Figure S1. For the valence band, the minimum bandwidth of 0.5 meV is attained at a strength of the electric field of 0.78 V/Å. For the conduction band, a minimum bandwidth of 1.4 meV is achieved at a strength of the electric field of 0.82 V/Å.

**Figure 2:**
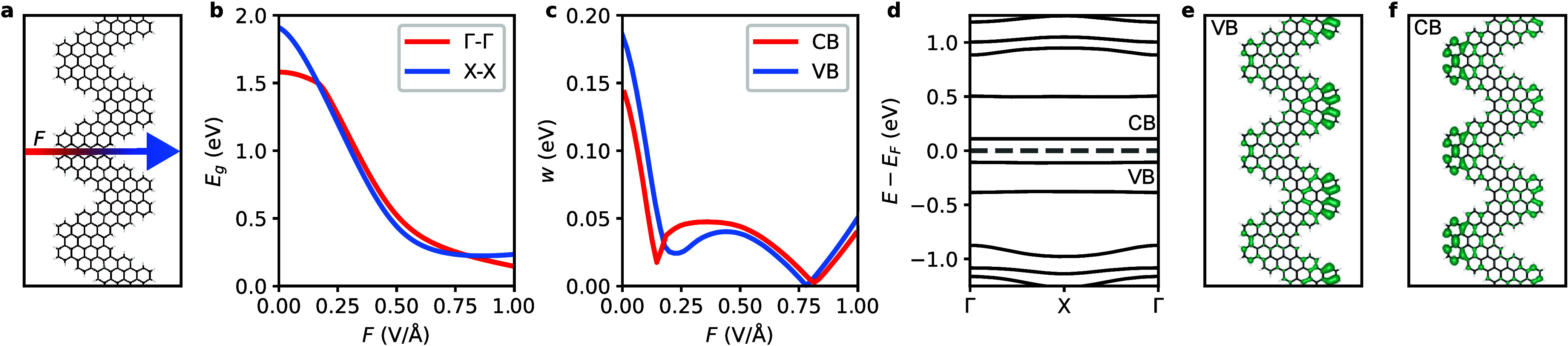
**Electric field-induced band flattening in CGNR. a**, Illustration of the external electric field (*F*) imposed in the direction transverse to the periodicity of the CGNR. Red and blue colors denote regions where the potential is higher and lower, respectively. **b**, Evolution of the band gap (*E*_*g*_) of the CGNR with *F*, as evaluated at the high-symmetry Γ and *X* points of the Brillouin zone. **c**, Evolution of the width (*w*) of the valence band (VB) and conduction band (CB) of the CGNR with *F*. **d**, Representative electronic band structure of the CGNR at *F* = 0.82 V/Å. The energy is referenced to the Fermi level (*E*_*F*_), marked by the horizontal dashed line. Local density of states (LDOS) of the **e**, VB of the CGNR at *F* = 0.78 V/Å and **f**, CB of the CGNR at *F* = 0.82 V/Å.

To gain insight into the spatial localization of these field-induced flat bands, we inspect their local density of states in Figure 2e,f. We observe that the electronic states associated with the valence and conduction bands are strictly confined at opposite edges of the CGNR. This localization effect can be attributed to the presence of edge protrusions in the atomic structure of the CGNR. Upon the application of the transverse electric field, these protrusions cause a nonuniform distribution of the potential at the carbon sites along the edges of the nanoribbon. Driven by the external field, the π-electrons (π-holes) of the CGNR localize at the protrusion, at which the potential is higher (lower), acting as quantum dots. It is noteworthy that this electric field-induced electron localization and the ensuing band flattening are unique features of CGNRs. In contrast, armchair and zigzag graphene nanoribbons, which lack protrusions, retain a uniform potential along each edge under the influence of an electric field. As a result, their band extrema preserve their dispersive character, as confirmed by earlier computational studies.^17,18^

*Doping-Induced Magnetism in CGNR*. The field-induced flat bands translate to a set of divergences in the electronic density of states, possibly rendering CGNR prone to electronic instabilities, resulting in magnetic phases when departing from charge neutrality. To verify this possibility, we investigated the response of CGNR to charge doping at the values of the electric field that minimize the valence and conduction bandwidths, respectively. In Figure 3a,d, we show the evolution of the magnetic moment of CGNR as a function of excess charge for both *p*-type and *n*-type doping, respectively. Upon doping, a finite magnetic moment arises when the excess charge exceeds ±0.25 |*e*|, reaching its maximum value of 1 μ_*B*_ at around ±1.0 |*e*| and vanishing at ±2.0 |*e*|. These findings reveal the emergence of a reversible, electrically induced magnetic phase transition in the CGNR. As shown in Supporting Information Figures S2 and S3, the doping-induced magnetism in CGNRs can arise at other electric field strengths, but the values of induced magnetic moments are maximized for the electric fields that minimize the valence or conduction bandwidth.

**Figure 3:**
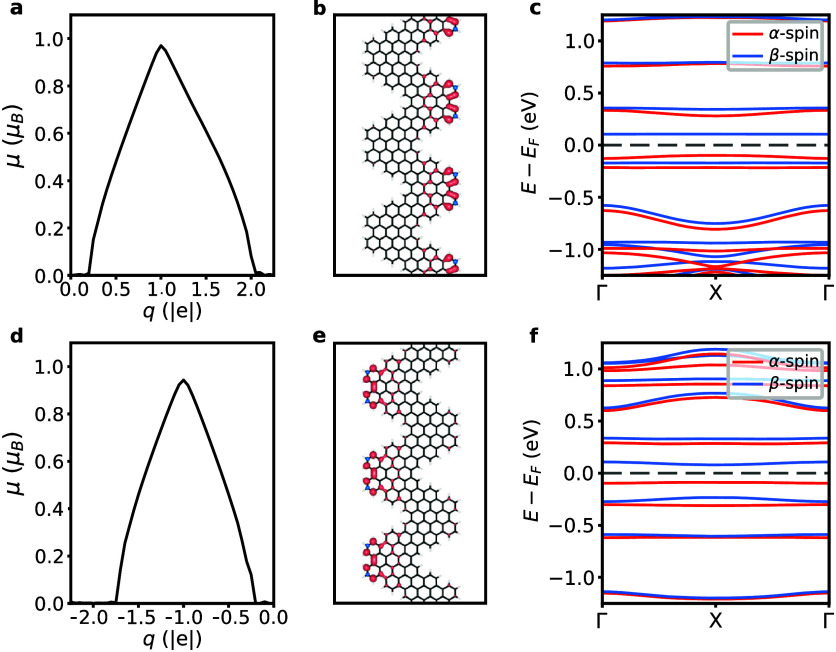
**Doping-induced magnetism in CGNR in a transverse electric field. a**, Evolution of the magnetic moment per unit cell (μ) with the excess charge (*q*) in the *p*-type doped CGNR at an external electric field *F* = 0.78 V/Å. **b**, Resulting spin-density of the CGNR at *F* = 0.78 V/Å and *q* = 1 |*e*|. **c**, Spin-resolved electronic band structure of the CGNR at *F* = 0.78 V/Å and *q* = 1 |*e*|. Red and blue lines denote α-spin and β-spin bands, respectively. The energy is referenced to the Fermi level (*E*_*F*_), marked by the horizontal dashed line. **d**, Evolution of μ with *q* in the *n*-type doped CGNR at *F* = 0.82 V/Å. **e**, Resulting spin density of the CGNR at *F* = 0.82 V/Å and *q* = −1 |*e*|. **f**, Spin-resolved electronic band structure of the CGNR at *F* = 0.82 V/Å and *q* = −1 |*e*|.

We inspect the spatial distribution of these magnetic moments by determining the spin density, namely, the difference in charge density between α-spin and β-spin charge densities. Figure 3b,e indicates a localization pattern of the spin density that is reminiscent of the local density of states (cf. Figure 2e,f) in that it primarily resides at protrusions along the edges of the nanoribbon. This gives rise to a ferromagnetic, one-dimensional spin-1/2 chain embedded in the CGNR. Remarkably, the localization of the magnetic moments on either edge of the nanoribbon is dictated by whether *n*- or *p*-type doping is introduced, pointing to a spatial control of magnetism in CGNR via the sign of the excess charge.^19^ In Figure 3c,f, we present the electronic band structure of the CGNR in the electrically induced magnetic phase. The valence and conduction bands are selectively contributed by α-spin and β-spin electrons, respectively, thus promoting a full spin-polarization of charge carriers around the Fermi level.

We suggest that the formation of the magnetic ground state in doped CGNR can be ascribed to a Stoner-like instability.^20^ According to the Stoner model, the presence or absence of magnetism in a system with a partially filled band is shaped by a trade-off between the exchange energy gain and the kinetic energy cost occurring upon spin polarization. This leads to the Stoner criterion, which can be used to ascertain whether a magnetic ground state is favored over a nonmagnetic one, through the relationship
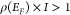
1where ρ(*E*_*F*_) is the electronic density of states of the nonmagnetic ground state at the Fermi level (*E*_*F*_) and *I* is the Stoner parameter. For the representative case of the *p*-type doped CGNR under the critical electric field, we estimate both ρ(*E*_*F*_) and *I* for varying excess charge *q*.

First, we determine the electronic density of states of doped CGNR under the electric field in the nonmagnetic state, which, as shown in Figure 4a, is found to exhibit a divergence centered at the Fermi level. Second, we determine the Stoner parameter, which connects the total energy *E* to the magnetic moment μ as
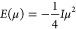
2To obtain the Stoner parameter *I*, we rely on the Landau theory, which provides the free energy as an expansion of the magnetic moment,^21^
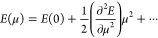
3where the Stoner parameter is related to the second-order term of the above expression as
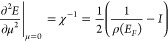
4with χ being the magnetic susceptibility. In practice, we (i) determine *E*(μ) through a set of *ab initio*, magnetic moment-constrained calculations, (ii) fit the resulting points with the expression given in equation 3, leading to the results given in Figure 4b, and (iii) extract *I* from the expression of the susceptibility given in equation 4.

**Figure 4:**
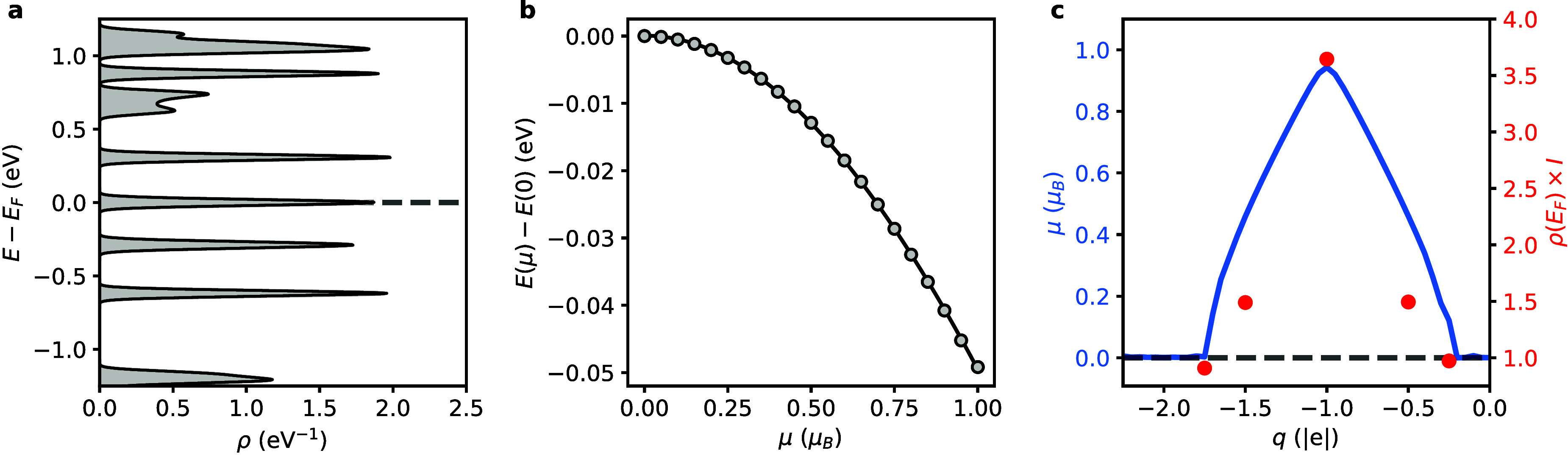
**Stoner-like magnetic instability in CGNR. a**, Electronic density of states (ρ) in the nonmagnetic phase of the CGNR at an external electric field *F* = 0.82 V/Å and excess charge *q* = −1 |*e*|. **b**, Evolution of the spin polarization energy (i.e., the difference in the total energy of the CGNR at finite and zero magnetic moments per unit cell, μ) with μ at *F* = 0.82 V/Å and *q* = −1 |*e*|. **c**, Product of the Stoner parameter (*I*) and ρ evaluated at the Fermi level (red points), along with μ (blue line) at *F* = 0.82 V/Å as a function of *q*.

In Figure 4c, we display the product ρ(*E*_*F*_) × *I* as a function of excess charge (the corresponding magnetic moments are also given for reference). We observe that the onset of the magnetic phase matches the Stoner criterion given in equation 1. Doping regions with finite magnetic moments are characterized by ρ(*E*_*F*_) × *I* > 1, whereas doping regions with vanishing magnetic moments are characterized by ρ(*E*_*F*_) × *I* < 1. This suggests that the observed doping-induced magnetism in CGNR can be quantitatively interpreted in terms of a Stoner-like instability.

*Simple Model Hamiltonian*. Finally, we propose a simple one-orbital, mean-field Hubbard model Hamiltonian to capture the essential physics of the electronic and magnetic properties of the CGNR. The model Hamiltonian involves only the unhybridized *p*_*z*_ orbitals of the *sp*^2^-bonded carbon atoms, reading
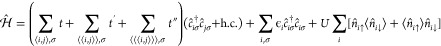
5where *t*, *t*′, and *t*″ are the first, second, and third nearest-neighbor hopping amplitudes, respectively, *ĉ*_*iσ*_^†^ (*ĉ*_*iσ*_) is the creation (annihilation) operator of the *p*_*z*_-electron with spin σ located at the *i*-th site (h.c. denotes the Hermitian conjugate), ϵ_*i*_ is the on-site potential at the *i*-th site, *n̂*_*iσ*_ = *ĉ*_*iσ*_^†^*ĉ*_*iσ*_ is the spin density at the *i*-th site, and *U* is the on-site Coulomb repulsion between a pair of *p*_*z*_-electrons located at the same site *i*. We use *t* = −2.75 eV, *t*′ = 0.22 eV, and *t*″ = −0.25 eV, as obtained from previous *ab initio* calculations,^22^ and *U* = 3.03 eV. This leads to a *U*-to-*t* ratio of 1.1,^23^ within the range of values estimated from *ab initio* methodologies (0.9 ≲ *U*/*t* ≲1.3 ^24^) and magnetic resonance experiments of neutral soliton states in a one-dimensional *sp*^2^ carbon polymer (1.1 ≲ *U*/*t* ≲ 1.3 ^25^). We verified that our results are qualitatively robust with respect to the choice of this parameter. To emulate the effect of the transverse electric field, we set the on-site potential to ϵ/2 at one edge and −ϵ/2 at the opposite edge, where the value of ϵ is determined by the strength of the applied electric field and the width of the CGNR, and linearly interpolate between these two values across the nanoribbon, as illustrated in Figure 5a.

**Figure 5:**
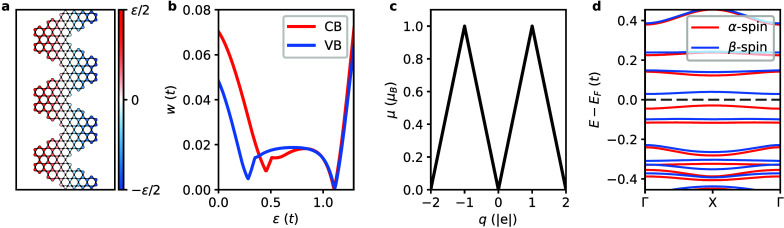
**Simple model Hamiltonian. a**, Schematic illustration of the distribution of the on-site energy (ϵ) adopted to emulate the external electric field. Red and blue colors denote sites where the potentials are higher and lower, respectively. **b**, Evolution of bandwidth (*w*) with the ϵ for the valence band (VB) and conduction band (CB). **c**, Evolution of the total magnetic moment per unit cell (μ) with the excess charge (*q*) for both *n*-type (*q* < 0) and *p*-type doping (*q* > 0) at ϵ = 1.09 *t*. **d**, Spin-resolved electronic band structure of the CGNR at ϵ = 1.09 *t* and *q* = −1 |*e*|. Red and blue lines denote α-spin and β-spin bands, respectively. The energy is referenced to the Fermi level (*E*_*F*_), marked by the horizontal dashed line.

We solve this mean-field Hubbard model Hamiltonian self-consistently.^19^ In Figure 5b, we display the evolution of the valence and conduction bandwidths with on-site potential ϵ. The observed trend closely resembles that obtained from *ab initio* results (cf. Figure 2c), with bandwidths steadily decreasing with ϵ in a nonmonotonic fashion. We then fix ϵ to the value that minimizes the valence and conduction bandwidths (i.e., 1.09*t*) and introduce charge doping. Albeit more symmetric, the evolution of magnetic moments with excess charge reported in Figure 5c is similar to that obtained from *ab initio* (cf. Figure 3a,d), with the maximum value of the magnetic moment developing at excess charge of ±1 |*e*|, leading to the electronic band structure shown in Figure 5d which features fully spin-polarized, ultraflat bands around the Fermi level. Despite its simplicity, our model Hamiltonian successfully reproduces the main features of the *ab initio* results, thus providing an intuitive understanding of the key physical quantities at play.

*Summary and Conclusions*. We have predicted the emergence of electrically induced ultraflat bands and π-electron magnetism in chevron graphene nanoribbons using *ab initio* calculations based on density functional theory. Our results indicate that the application of a transverse electric field leads to the formation of isolated, perfectly flat bands featuring a dispersion of ∼1 meV in the vicinity of the Fermi level which originates from the confinement of charge carriers at the protrusions that characterize the edges of these nanoribbons. Upon charge doping, these flat bands undergo a Stoner-like electronic instability, leading to the development of an array of local magnetic moments at the edges, acting as a one-dimensional spin-1/2 chain hosted in the nanoribbon. We have additionally proposed a simple mean-field Hubbard model Hamiltonian to capture the essential physical effects that are operative in the formation of the correlated electronic phases. To conclude, our findings expand the class of graphene nanostructures hosting flat bands beyond moiré systems and envision new opportunities to design reversible, strongly correlated electronic states in chevron graphene nanoribbons, with possible applications in spintronics and related quantum devices.

## Supporting Information Available

Additional first-principles calculations, including the evolution of the electronic band structure and magnetic moments of chevron graphene nanoribbons with the external electric field (PDF)

## References

[ref1] Andrei E. Y.; MacDonald A. H. Graphene bilayers with a twist. Nat. Mater. 2020, 19, 1265 10.1038/s41563-020-00840-0. 33208935

[ref2] Bistritzer R.; MacDonald A. H. Moiré bands in twisted double-layer graphene. Proc. Natl. Acad. Sci. U. S. A. 2011, 108, 12233–12237. 10.1073/pnas.1108174108. 21730173 PMC3145708

[ref3] Lisi S.; Lu X.; Benschop T.; de Jong T. A.; Stepanov P.; Duran J. R.; Margot F.; Cucchi I.; Cappelli E.; Hunter A.; Tamai A.; Kandyba V.; Giampietri A.; Barinov A.; Jobst J.; Stalman V.; Leeuwenhoek M.; Watanabe K.; Taniguchi T.; Rademaker L.; et al. Observation of flat bands in twisted bilayer graphene. Nat. Phys. 2021, 17, 189 10.1038/s41567-020-01041-x.

[ref4] Tarnopolsky G.; Kruchkov A. J.; Vishwanath A. Origin of Magic Angles in Twisted Bilayer Graphene. Phys. Rev. Lett. 2019, 122, 106405 10.1103/PhysRevLett.122.106405. 30932657

[ref5] Cao Y.; Fatemi V.; Demir A.; Fang S.; Tomarken S. L.; Luo J. Y.; Sanchez-Yamagishi J. D.; Watanabe K.; Taniguchi T.; Kaxiras E.; Ashoori R. C.; Jarillo-Herrero P. Correlated insulator behaviour at half-filling in magic-angle graphene superlattices. Nature 2018, 556, 80 10.1038/nature26154. 29512654

[ref6] Cao Y.; Fatemi V.; Fang S.; Watanabe K.; Taniguchi T.; Kaxiras E.; Jarillo-Herrero P. Unconventional superconductivity in magic-angle graphene superlattices. Nature 2018, 556, 43 10.1038/nature26160. 29512651

[ref7] Sharpe A. L.; Fox E. J.; Barnard A. W.; Finney J.; Watanabe K.; Taniguchi T.; Kastner M. A.; Goldhaber-Gordon D. Emergent ferromagnetism near three-quarters filling in twisted bilayer graphene. Science 2019, 365, 605 10.1126/science.aaw3780. 31346139

[ref8] Schleder G. R.; Pizzochero M.; Kaxiras E. One-Dimensional Moiré Physics and Chemistry in Heterostrained Bilayer Graphene. J. Phys. Chem. Lett. 2023, 14, 8853 10.1021/acs.jpclett.3c01919. 37755819

[ref9] Ghorashi S. A. A.; Dunbrack A.; Abouelkomsan A.; Sun J.; Du X.; Cano J. Topological and Stacked Flat Bands in Bilayer Graphene with a Superlattice Potential. Phys. Rev. Lett. 2023, 130, 196201 10.1103/PhysRevLett.130.196201. 37243639

[ref10] Cai J.; Ruffieux P.; Jaafar R.; Bieri M.; Braun T.; Blankenburg S.; Muoth M.; Seitsonen A. P.; Saleh M.; Feng X.; Müllen K.; Fasel R. Atomically precise bottom-up fabrication of graphene nanoribbons. Nature 2010, 466, 470 10.1038/nature09211. 20651687

[ref11] Chen Z.; Narita A.; Müllen K. Graphene nanoribbons: On-surface synthesis and integration into electronic devices. Adv. Mater. 2020, 32, 2001893 10.1002/adma.202001893. 32945038

[ref12] Perdew J. P.; Burke K.; Ernzerhof M. Generalized gradient approximation made simple. Phys. Rev. Lett. 1996, 77, 3865 10.1103/PhysRevLett.77.3865. 10062328

[ref13] Soler J. M.; Artacho E.; Gale J. D.; García A.; Junquera J.; Ordejón P.; Sánchez-Portal D. The SIESTA method for *ab initio* order-*N* materials simulation. J. Phys.: Condens. Matter 2002, 14, 2745 10.1088/0953-8984/14/11/302. 21693870

[ref14] Troullier N.; Martins J. L. Efficient pseudopotentials for plane-wave calculations. Phys. Rev. B 1991, 43, 1993 10.1103/PhysRevB.43.1993. 9997467

[ref15] Vo T. H.; Shekhirev M.; Kunkel D. A.; Morton M. D.; Berglund E.; Kong L.; Wilson P. M.; Dowben P. A.; Enders A.; Sinitskii A. Large-scale solution synthesis of narrow graphene nanoribbons. Nat. Commun. 2014, 5, 3189 10.1038/ncomms4189. 24510014

[ref16] Bronner C.; Marangoni T.; Rizzo D. J.; Durr R. A.; Jørgensen J. H.; Fischer F. R.; Crommie M. F. Iodine versus Bromine Functionalization for Bottom-Up Graphene Nanoribbon Growth: Role of Diffusion. J. Phys. Chem. C 2017, 121, 18490–18495. 10.1021/acs.jpcc.7b02896.

[ref17] Pizzochero M.; Tepliakov N. V.; Mostofi A. A.; Kaxiras E. Electrically induced Dirac fermions in graphene nanoribbons. Nano Lett. 2021, 21, 9332 10.1021/acs.nanolett.1c03596. 34714095

[ref18] Son Y.-W.; Cohen M. L.; Louie S. G. Half-metallic graphene nanoribbons. Nature 2006, 444, 347 10.1038/nature05180. 17108960

[ref19] Tepliakov N. V.; Ma R.; Lischner J.; Kaxiras E.; Mostofi A. A.; Pizzochero M. Dirac Half-Semimetallicity and Antiferromagnetism in Graphene Nanoribbon/Hexagonal Boron Nitride Heterojunctions. Nano Lett. 2023, 23, 6698–6704. 10.1021/acs.nanolett.3c01940. 37459271

[ref20] Gao S.; Yang L. Edge-insensitive magnetism and half metallicity in graphene nanoribbons. J. Phys.: Condens. Matter 2018, 30, 48LT01 10.1088/1361-648X/aae9cb. 30406766

[ref21] Mohn P. Magnetism in the Solid State: An Introduction; Springer-Verlag: Berlin, 2003.

[ref22] Tepliakov N. V.; Lischner J.; Kaxiras E.; Mostofi A. A.; Pizzochero M. Unveiling and Manipulating Hidden Symmetries in Graphene Nanoribbons. Phys. Rev. Lett. 2023, 130, 026401 10.1103/PhysRevLett.130.026401. 36706398

[ref23] Yazyev O. V. Emergence of magnetism in graphene materials and nanostructures. Rep. Prog. Phys. 2010, 73, 056501 10.1088/0034-4885/73/5/056501.

[ref24] Pisani L.; Chan J. A.; Montanari B.; Harrison N. M. Electronic structure and magnetic properties of graphitic ribbons. Phys. Rev. B 2007, 75, 064418 10.1103/PhysRevB.75.064418.

[ref25] Thomann H.; Dalton L. K.; Grabowski M.; Clarke T. C. Direct observation of Coulomb correlation effects in polyacetylene. Phys. Rev. B 1985, 31, 3141 10.1103/PhysRevB.31.3141. 9936180

