# Supplementary material for: Electrically Tunable Ultraflat Bands and π-Electron Magnetism in Graphene Nanoribbons
Source: J Phys Chem Lett. 2025 Feb 7;16(7):1680–5. doi: 10.1021/acs.jpclett.5c00121 (PMC11849038; doi:10.1021/acs.jpclett.5c00121)
Supplement: Supplementary file 1 — jz5c00121_si_001.pdf [file jz5c00121_si_001.pdf]

Supporting Information

Electrically tunable ultra-flat bands and π-electron magnetism in graphene nanoribbons

Ruize Ma,[†,‡,¶] Nikita V. Tepliakov,[‡,¶] Arash A. Mostofi,[‡,¶] and Michele Pizzochero[*,§,‖]

†*Department of Physics, ETH Zürich, Zurich 8093, Switzerland*

‡*Departments of Materials and Physics, Imperial College London, London SW7 2AZ, United Kingdom*

¶*The Thomas Young Centre for Theory and Simulation of Materials, Imperial College London, London SW7 2AZ, United Kingdom*

§*Department of Physics, University of Bath, Bath BA2 7AY, United Kingdom*

‖*School of Engineering and Applied Sciences, Harvard University, Cambridge, MA 02138, United States*

E-mail: mp2834@bath.ac.ak

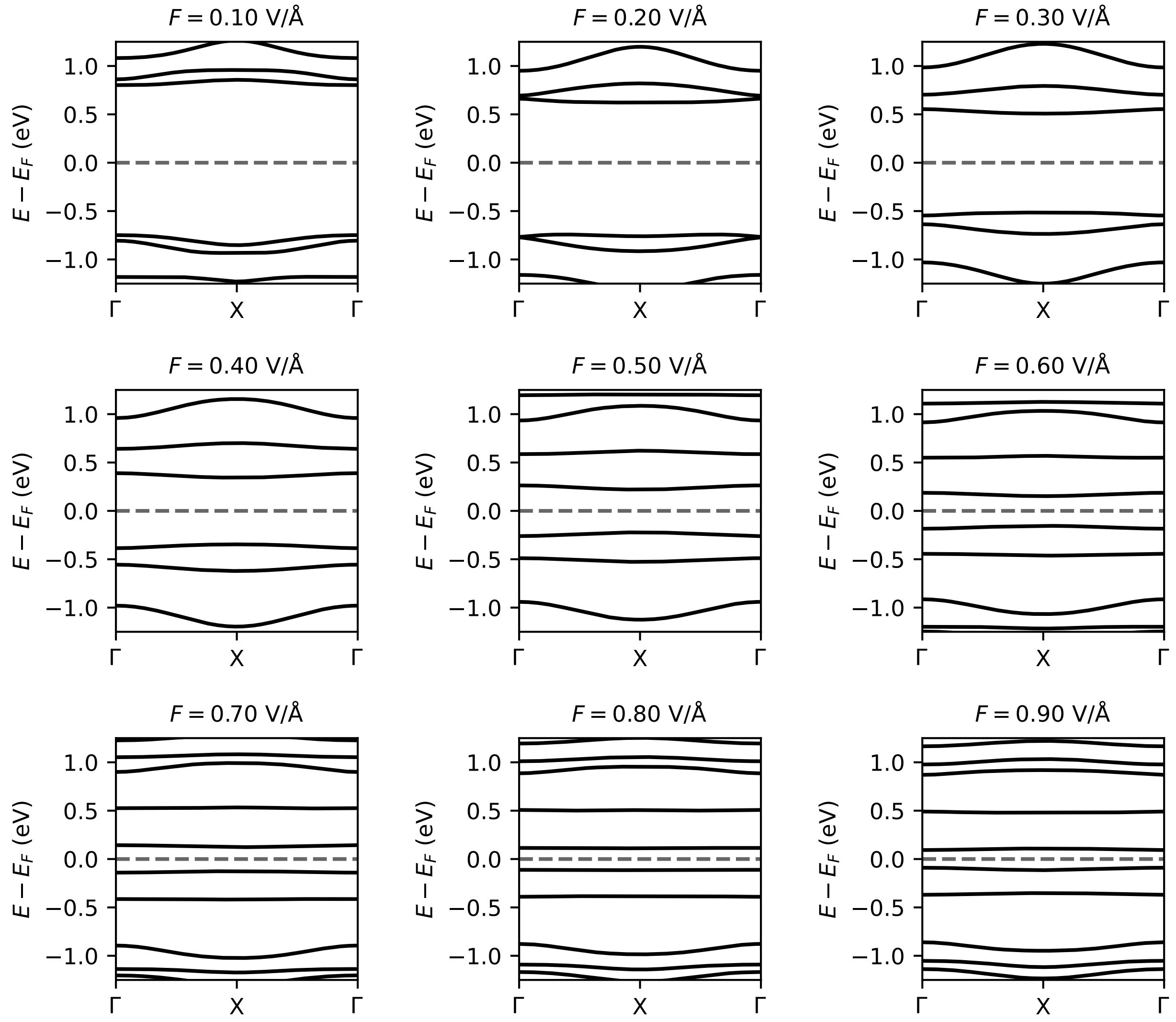

Supporting Figure S1. Evolution of the electronic band structure of the CGNR with the strength of the external electric field (F). The energy is referenced to the Fermi level (E_F), marked by the horizontal dashed line.

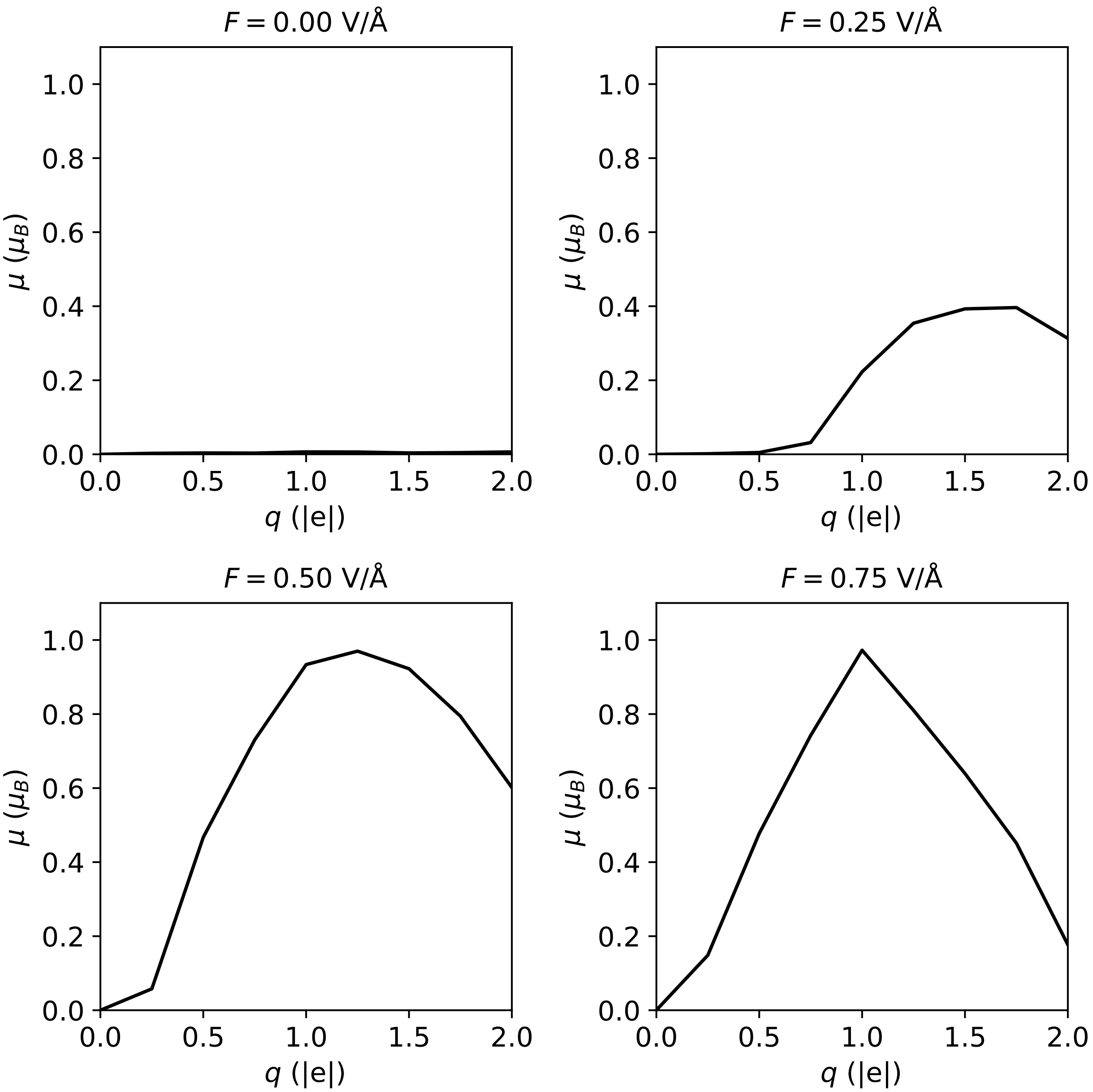

Supporting Figure S2. Evolution of the magnetic moment per unit cell (μ) with the excess charge (q) in the CGNR with the strength of the external electric field (F).

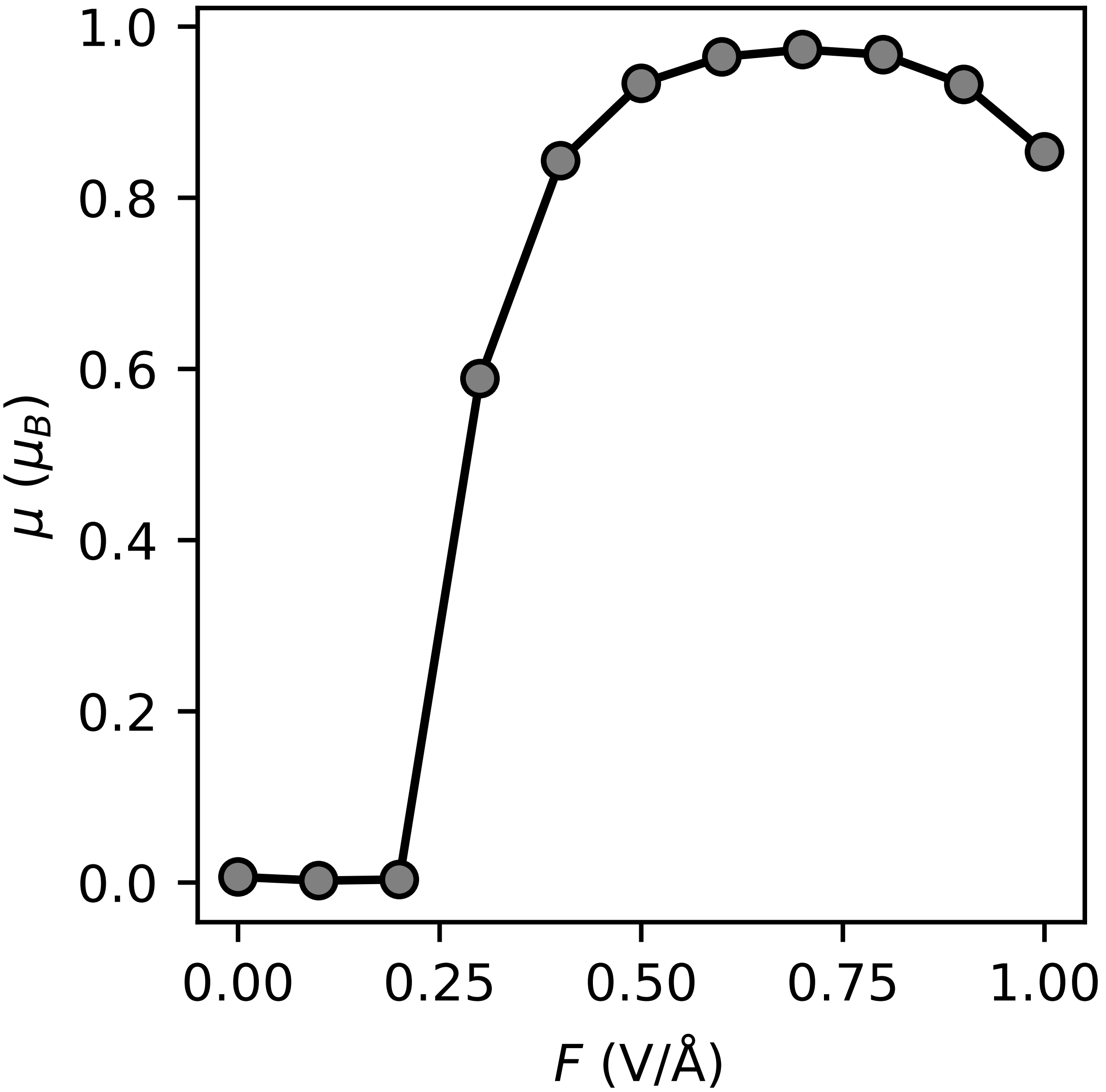

Supporting Figure S3. Evolution of the magnetic moment per unit cell (μ) with the strength of the external electric field (F) in the CGNR for a fixed value of excess charge q = 1.0 $|e|$.
